# Application of targeted diagnosis of PSMA in the modality shift of prostate cancer diagnosis: a review

**DOI:** 10.3389/fonc.2023.1179595

**Published:** 2023-09-01

**Authors:** Li Yan, Zhengchao Zhang, Ting Wang, Leihong Yuan, Xiaoke Sun, Pengxiao Su

**Affiliations:** Department of Urology, Honghui Hospital, Xi’an Jiaotong University, Xi’an, China

**Keywords:** prostate cancer, PSMA, PET, gallium-68, fluoro-18, mpMRI

## Abstract

Prostate cancer (PCa) is a serious threat to the health of men all over the world. The progression of PCa varies greatly among different individuals. In clinical practice, some patients often progress to advanced PCa. Therefore, accurate imaging for diagnosis and staging of PCa is particularly important for clinical management of patients. Conventional imaging examinations such as MRI and CT cannot accurately diagnose the pathological stages of advanced PCa, especially metastatic lymph node (LN) stages. As a result, developing an accurate molecular targeted diagnosis is crucial for advanced PCa. Prostate specific membrane antigen (PSMA) is of great value in the diagnosis of PCa because of its specific expression in PCa. At present, researchers have developed positron emission tomography (PET) targeting PSMA. A large number of studies have confirmed that it not only has a higher tumor detection rate, but also has a higher diagnostic efficacy in the pathological stage of advanced PCa compared with traditional imaging methods. This review summarizes recent studies on PSMA targeted PET in PCa diagnosis, analyzes its value in PCa diagnosis in detail, and provides new ideas for urological clinicians in PCa diagnosis and clinical management.

## 1 Introduction

Prostate cancer (PCa) is a serious threat to the global male health of public health events. PCa affects millions of men worldwide each year. In developed areas, the disease is one of the most common solid cancers, and its prognosis varies significantly based on factors such as age, ethnicity, genetic heritage, and stage of development (1, 2). PCa is the most common non-cutaneous malignancy among men in the United States, with an estimated 268,490 new cases in the United States in 2022 (3). The risk of PCa increases with age (2). As a result, PCa rates are p articularly high in areas with high life expectancy, such as United States and United Kingdom. The global incidence of PCa is positively correlated with the Human Development Index (HDI) and gross domestic product, so the incidence is generally higher in developed countries than in developing countries (4). PCa risk is strongly associated with a family history of any cancer, and the incidence in these families is thought to be the highest of all malignancies (5, 6). Furthermore, non-genetic factors thought to increase prostate cancer-related mortality include smoking, obesity, and a predominantly Western diet. However, there is a lack of evidence of an impact on disease incidence (7, 8). The overall aggressiveness of PCa varies considerably between individuals. Some patients will still progress to metastatic castration-resistant PCa (mCRPC) after formal treatment. Therefore, it is particularly important to select appropriate diagnosis and monitoring strategies for prostate cancer patients. Guidelines from the National Comprehensive Cancer Network (NCCN) stratify patients by risk based on serum prostate-specific antigen (PSA) levels, while staging patients based on imaging. The introduction of multi-parameter magnetic resonance imaging (mpMRI) has improved the diagnosis of PCa.

Current standard diagnostic strategies for PCa include digital rectal examination (DRE) and serum PSA level testing. If DRE is abnormal and/or serum PSA levels are elevated, systematic transrectal ultrasound (TRUS) biopsies are performed according to the Gleason grading system and the modified histological grading of the International Society of Urological Pathology (ISUP) to assess the presence of PCa (3, 9). In recent years, clinical studies have found that these diagnostic strategies lead to problems such as the risk of overdiagnosis and overtreatment of lazy tumors based on serum PSA levels, and the possibility of missing some anterior lesions when using TRUS biopsies (9). Over the past decade, mpMRI has become an important technique in the diagnosis and management of PCa. It is increasingly used for targeted biopsies, detection, disease staging, assessment of disease invasiveness, and patient follow-up after a negative biopsy (10–12). As an important diagnostic method for PCa, mpMRI has become the preferred diagnostic method for clinically suspected PCa patients and has been included in national and international guidelines (13). The prostate mpMRI imaging reporting and data system can stratify the probability of clinically significant PCa (csPCa) (14). However, this method also has certain limitations, namely absolute and relative contraindications, and cannot be used in patients with claustrophobia. On the other hand, despite the introduction of specific criteria for prostate imaging reporting and data systems (15, 16), mpMRI may still produce ambiguous results, with the possibility of underestimating the scale and scope and missing clinically significant lesions. MRI has also been used clinically to guide biopsy of suspicious prostate target areas in recent years (17). The MRI-guided targeted biopsy pathway has been shown to significantly improve PCa detection rate (DR) compared to conventional systematic biopsy (18, 19). Furthermore, improvements in MRI localization of PCa have facilitated the development of focal treatments such as cryotherapy and high-intensity focused ultrasound (20). Bone scintigraphy (BS) is the most widely used method for the early assessment of bone metastasis in PCa (21). There is a low specificity to BS imaging, despite its high sensitivity. There is a difficulty for BS in distinguishing metastases from bone tumors, trauma, degenerative changes, and infection.

**Figure 1:**
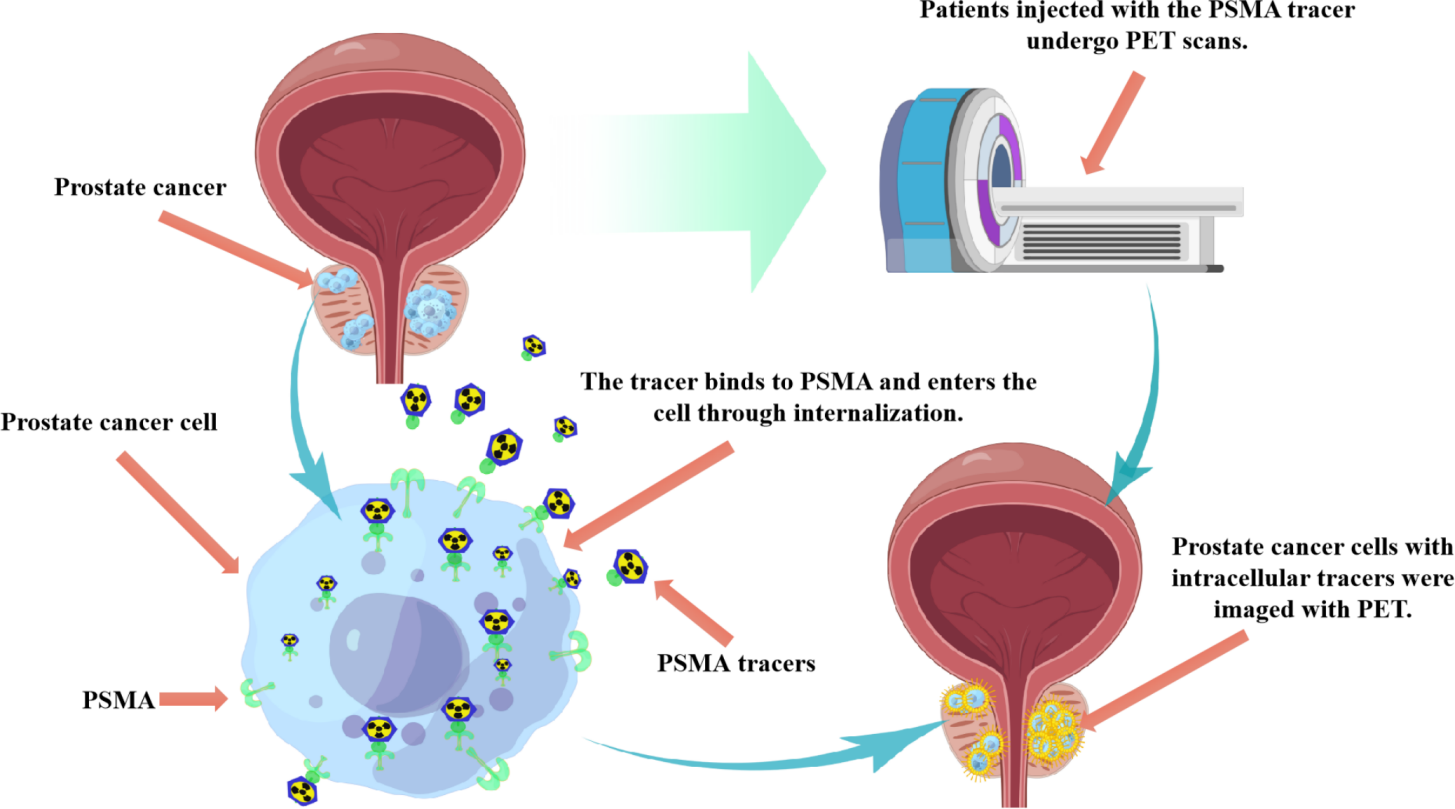
Schematic diagram of application principle of PSMA PET imaging technique in PCa diagnosis. The PSMA receptor binding protein on the cell surface promotes the concentration of labeled radioisotopes in the cell through internalization, thus achieving highly targeted visualization by PET.

Positron emission tomography (PET) imaging targeting prostate specific membrane antigen (PSMA), as a novel imaging method, has demonstrated its potential as an adjoint or alternative imaging technique for PCa diagnosis. PSMA is a type 2 transmembrane glycoprotein that has been found to be overexpressed in prostate tumors, and its expression levels are associated with high serum PSA levels and higher Gleason scores (22–24). PSMA protein is divided into three parts, namely intracellular part, transmembrane part and extracellular part (25, 26). The extracellular portion makes up 95% of the PSMA protein and is an accessible target for small molecules and antibody-based drugs used in imaging and therapy. The PSMA receptor binding protein on the cell surface promotes the concentration of labeled radioisotopes in the cell through internalization, thus achieving highly targeted visualization by PET (**Figure 1**). This is also the molecular basis of PSMA ligand molecular imaging for PCa diagnosis. Furthermore, studies have shown that the expression of PSMA is increased in 85-100% PCa tissues, especially in metastatic PCa (27). PSMA is also expressed in the healthy prostate, small intestine, central nervous system, proximal renal tubules, salivary glands, and lacrimal glands. However, the expression of PSMA in PCa cell membranes was 10-1000 times higher than that in normal cells, and further studies showed that the expression increased with the increase of PCa stage and grade (28, 29). PSMA expression was strongly correlated with Gleason score and serum PSA value (30). During androgen deprivation therapy, it was found that the expression of PSMA was regulated by androgen receptor (31). Interestingly, PSMA expression levels were negatively correlated with androgen levels. Therefore, the diagnosis of targeted PSMA can be applied in conditions with low androgen activity, such as castration-resistant PCa (CRPC) (32–34).Therefore, PSMA can be used as a targeted and specific marker in the targeted diagnosis and treatment of PCa (35, 36).

PSMA has become an attractive diagnostic target for small molecular ligands in PCa. It can be tagged with a positron emitter for PET imaging (37). PET/computed tomography (PET/CT) targeting PSMA for PCa staging is increasingly used worldwide (38). As a new diagnostic method, PET/CT of PSMA ligand enhances the power of accurate diagnosis and pathological staging of patients with advanced PCa (39). A number of clinical trials are underway to study the effectiveness of PSMA as a diagnostic tool. PSMA binding ligands (including antibodies or small molecules) are labeled with radionuclide tracers and can be used in the diagnosis of PCa. Currently, gallium-68 (68Ga) and fluoro-18 (18F) are the most widely used tracers (40). PSMA binding radionuclides can be internalized into tumor cells, and then highly targeted visualization can be achieved by PET (41). This review summarizes the role of PSMA targeted diagnosis in PCa diagnosis and its influence on prognosis, providing a new PCa diagnosis idea for clinicians and optimizing the clinical management of PCa patients.

## 2 Materials and methods

A systematic review was conducted in accordance with the preferred reporting items for systematic reviews guidelines (PRISMA). The authors ran queries to retrieve prospective and retrospective studies on the use of radiomic analysis of PSMA targeted PET in PCa diagnosis in the most relevant databases and web sources (PubMed and Web of Science). We pooled the terms(“PSMA”), (“prostate cancer”, “prostatic neoplasms”) and (“radioligand”, “radiotracer”) using the Boolean operator. English-language original articles published before April 2023 were considered. The included published articles were all clinical studies reporting the use of PSMA ligands for imaging of PCa patients. Preclinical studies, case reports and abstracts were not considered. Given the heterogeneity in terms of disease characteristics, clinical context and the absence of randomized controlled trials, no meta-analysis was performed. The Methodological Index for Non-Randomized Studies (MINORS) score was used by two reviewers to evaluate the quality of all included non-randomized studies (42). We defined PSMA-PET results as the index test and histopathology findings as the reference standard.

## 3 Results

### 3.1 Study selection

**Figure 2:**
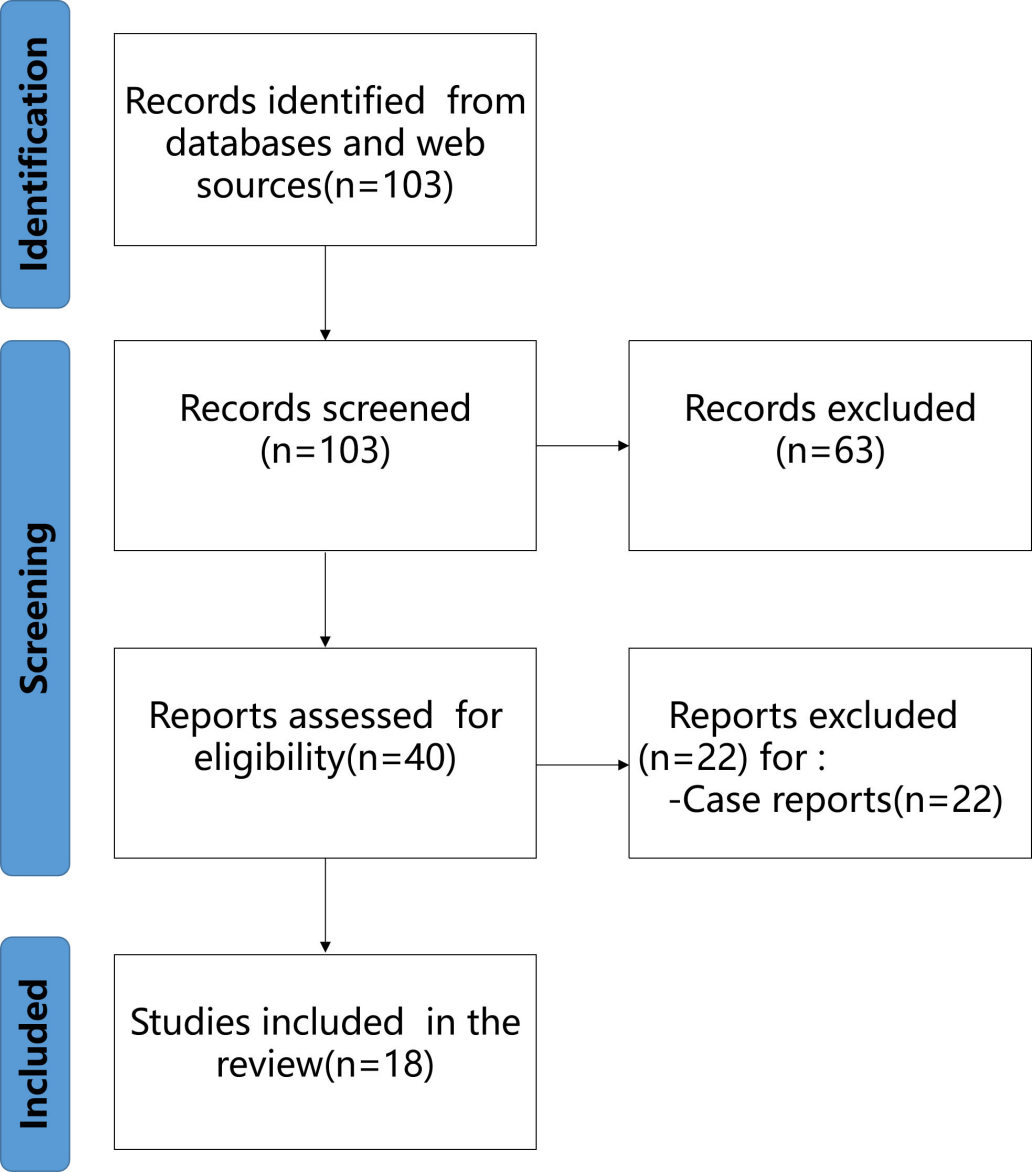
The systematic review flow diagram of selected studies.

The selection flow diagram adapted from the PRISMA recommendations is illustrated in **Figure 2**. We included 18 studies that explored the value of PSMA radioligands for prostate cancer diagnosis. Among them, there were 12 studies on the correlation of PSMA radioligands with different tracers, and 6 comparative correlation studiesstudies between the diagnostic value of PSMA radioligands and traditional diagnostic methods. Based on the MINORS criteria (**Table 1**), the mean study quality score was 16.4 ± 3.5. For the comparative studies, the mean MINORS score was 18.8 ± 0.8. For the 4 non-comparative studies, the mean MINORS score was 11.7 ± 0.9. A total of 8 high-quality studies, and a further 10 intermediate-quality studies were identified.

**Table 1:** Quality assessment of included non-randomized studies.

Authors	Clearly Stated Aim	Inclusion of Consecutive Patients	Prospective Collection of Data	Endpoints Appropriate for Aim	Unbiased Assessment of Endpoints	Appropriate follow-up Period	Lost to Follow-up <5%	Prospective Calculation of Study Size	Adequate Control Group	Contemporary Groups	Baseline Equivalence of Groups	Adequate Statistical Analysis	Total score
Rowe SP et al., 2016 (43)	2	2	0	2	2	2	2	0	0	0	0	1	13
Dietlein F et al., 2017 (44)	2	2	0	2	2	1	2	0	0	0	0	0	11
Giesel FL et al.2017 (45)	2	2	0	2	2	2	2	0	2	2	2	1	19
Calais J et al.2018 (46)	2	2	0	2	1	2	2	0	2	2	1	2	18
Calais J et al.2019 (47)	2	2	0	2	2	2	2	0	2	2	1	1	18
Ceci F et al.2019 (48)	2	2	0	2	2	1	2	0	0	0	0	1	12
Witkowska-Patena E et al.2019 (49)	2	2	0	1	2	1	2	0	2	2	2	2	18
Fendler WP et al.2020 (50)	2	2	0	2	2	1	2	0	0	0	0	0	11
Song H et al.2020 (51)	2	2	0	2	2	1	2	0	0	0	0	1	12
Jansen BHE et al.2021 (52)	2	2	0	1	2	1	2	1	0	0	0	0	11
Matushita CS et al.2021 (53)	2	2	0	2	2	2	2	0	2	2	2	2	20
Malaspina S et al.2022 (54)	2	2	0	2	2	2	2	0	2	2	2	2	20
Pyka T et al.2016 (55)	2	2	0	2	2	2	2	0	2	2	2	1	19
Rhee H et al.2016 (56)	2	2	0	2	1	2	2	1	2	2	1	2	19
Tulsyan S, et al.2017 (57)	2	2	0	2	2	1	2	0	2	2	1	2	18
Nandurkar R et al.2019 (58)	2	2	0	2	1	2	2	0	2	2	2	2	19
Zhang LL et al.2021 (59)	2	2	0	2	2	2	2	0	2	2	2	1	19
Szigeti F et al.2022 (60)	2	2	0	1	2	1	2	0	2	2	2	2	18

### 3.2 Application of PET imaging targeting PSMA in PCa diagnosis

In recent years, PET imaging targeting PSMA has made great progress in the diagnosis of PCa. A large number of studies have been conducted in this field, mainly detecting and comparing the diagnostic efficacy of different targeted PSMA tracers (**Table 2**). Studies have shown that PET imaging targeting PSMA has higher accuracy, specificity and sensitivity than traditional imaging (53, 61). It can not only be used in the screening of primary PCa, but also in the pathological staging of recurrent or metastatic PCa (43, 62–64).

**Table 2:** Studies on the application of PET targeted PSMA in the diagnosis of PCa.

Interventions	Type of Study	Objectives	Number of Studies and/or Patients	Conclusion	References
18F-DCFPyL- PSMA PET/CT	Prospective non-randomized controlled study	This study evaluated the diagnostic efficacy of 18F-PSMA PET/CT in patients with metastatic PCa.	18 patients	PCa sites can be detected with 18F-PSMA PET/CT in large numbers.	Rowe SP et al., 2016 (43)
18F-PSMA PET and 68Ga-PSMA PET	Retrospective non-randomized controlled study	In this study, 18F-PSMA PET and 68Ga-PSMA PET were evaluated for diagnostic efficiency.	191 patients	As compared to 68Ga-PSMA PET, 18F-PSMA PET has comparable diagnostic efficiency.	Dietlein F et al., 2017 (44)
18F-PSMA-1007	Prospective non-randomized controlled study	This study compared the diagnostic performance of 18F-PSMA-1007 with 68Ga-PSMA-11 in human volunteers and patients.	13 patients	There is no difference in performance between 18F-PSMA-1007 and 68Ga-PSMA-11.	Giesel FL et al.2017 (45)
68Ga- PSMA-11 and 18F-fluciclovine PET/CT	Retrospective clinical study	A comparative study was conducted between 18F-fluciclovine PET/CT and 68Ga-PSMA-11 PET/CT for PCa diagnosis.	10 patients	The recurrence rate of PCa was higher with 68Ga-PSMA-11 PET/CT than with 18F-fluluciclovine PET/CT.	Calais J et al.2018 (46)
18F–fluiclovine and PSMA PET-CT	Prospective non-randomized controlled study	The objective of this study was to compare the diagnostic efficacy of 18F- fluiclovine and PSMA PET-CT in biochemical recurrent PCa.	50 patients	The DR of PSMA PET-CT imaging is higher for PCa patients with low PSA concentration and BCR after radical prostatectomy.	Calais J et al.2019 (47)
68Ga-PSMA PET/CT	Prospective non-randomized controlled study	68Ga-PSMA PET/CT DR for PCa recurrence sites was evaluated in this study.	332 patients	PCa patients with BCR benefit from 68Ga-PSMA-11 PET/CT.	Ceci F et al.2019 (48)
F-Choline PET/CT and 18F-PSMA-1007 PET/CT	Prospective non-randomized controlled study	This study examined the performance of F-Choline PET/CT and 18F-PSMA-1007 PET/CT in patients with biochemical recurrent PCa.	40 patients	The DR of 18F-PSMA-1007 was higher than that of F-Choline PET/CT.	Witkowska-Patena E et al.2019 (49)
68Ga-PSMA PET	Prospective multicenter non-randomized controlled study	This study examined whether 68Ga-PSMA PET could be used to treat recurrent PCa.	635 patients	More than half of patients with BCR of PCa can be identified by 68Ga-PSMA PET.	Fendler WP et al.2020 (50)
18F-PSMA PET/CT	Prospective non-randomized controlled study	In this study, 18F-PSMA PET/CT was evaluated in patients with BCR of PCa.	72 patients	Patients with 18F-PSMA PET/CT have a high positive rate and their clinical treatment is affected by it in 60% of the cases.	Song H et al.2020 (51)
18F-PSMA PET/CT	Prospective, multicenter non-randomized controlled study	This study evaluated the diagnostic value of 18F-PSMA PET/CT in LN staging of primary PCa.	117 patients	18F-PSMA PET/CT showed high specificity, but limited sensitivity.	Jansen BHE et al.2021 (52)
68Ga-PSMA PET	Prospective and retrospective cross-sectional studies	This study assessed the accuracy of 68Ga-PSMA PET in the diagnosis of PCa.	35 studies/3910 patients	In the diagnosis of PCa, 68Ga-PSMA PET has greater sensitivity and specificity than conventional imaging.	Matushita CS et al.2021 (53)
18F-rhPSMA-7.3 PET/CT	Prospective controlled study	This study evaluated the efficacy of 18F-rhPSMA-7.3 PET/CT in prostate cancer patients.	10 patients	PCa primary and metastases were detected well using 18F-rhPSMA-7.3 PET/CT.	Malaspina S et al.2022 (54)

#### 3.2.1 68Ga-PSMA PET

68GA-labeled PSMA PET (68Ga-PSMA PET) is an emerging imaging method that has been shown to be of great value in the diagnosis of PCa by numerous studies. 68GA-PSMA-11 was approved by the U.S. Food and Drug Administration (FDA) in 2020 as the first 68Ga radiopharmaceutical for PET imaging of PSMA-positive PCa (65). This radiopharmaceutical can bind to PSMA of PCa, which in turn allows specific imaging of tumor cells. Corfield J et al. (66) systematically evaluated the application value of 68Ga-PSMA PET in the primary stage of high-risk PCa. The study included a study of 68Ga-PSMA PET for primary staging of PCa. The results showed that the DR of malignant lesions of 68Ga-PSMA PET was significantly higher than that of conventional imaging mode. Similarly, Hu X et al. (62) discussed the feasibility of 68Ga-PSMA PET/CT in the diagnosis of primary PCa. The results showed that 68Ga-PSMA PET/CT had higher sensitivity and specificity than conventional imaging in the diagnosis of primary PCa. Based on its advantages of high DR, 68Ga-PSMA PET/CT should be popularized in the detection of primary PCa. von Eyben FE et al. (67) evaluated the DR and diagnostic accuracy of 68Ga-PSMA PET/CT for PCa through a meta-analysis. The study found that 68Ga-PSMA PET/CT is of clinical significance in detecting the recurrence site of PSA patients with PSA<1.0ng/ml after radical prostatectomy. Matushita CS et al. (53) discussed the accuracy of 68Ga-PSMA PET in the diagnosis of PCa through a cross-sectional study. The summary sensitivity and specificity were 0.90 and 0.90, respectively. For the staging of PCa, the pooled sensitivity and specificity were 0.93 and 0.96, respectively. This study confirmed that 68Ga-PSMA PET has higher sensitivity and specificity in the diagnosis of PCa than conventional imaging. Therefore, the above studies suggest that 68Ga-PSMA PET/CT as a non-invasive diagnostic tool can be applied to PCa with PSMA expression and more accurately assess disease staging.

PCa metastasis and recurrence after treatment is one of the major challenges urologists facing. Management of metastasis and recurrence depends on site and burden. Therefore, it is urgent to develop an early imaging technique to accurately locate metastatic and recurrent lesions. It was found that 68Ga-PSMA PET also has high diagnostic value in PCa metastasis and recurrence stage. Hope TA et al. (68) investigated the diagnostic sensitivity and specificity of 68Ga-PSMA-11 PET in the initial staging and biochemical recurrence (BCR) of metastatic PCa through a meta-analysis. LN pathology during radical prostatectomy was used as the gold standard. The sensitivity and specificity of 68Ga-PSMA-11 in the initial stage diagnosis of PCa were 0.74 and 0.96, respectively. For BCR, the positive predictive value was 0.99. When PSA<2.0, the DR was 0.63. When PSA>2.0, the DR was 0.94. This study suggests that 68Ga-PSMA-11 has good localization value in the initial staging and BCR of metastatic PCa. Eissa A et al. (69) evaluated the role of 68Ga-PSMA PET/CT scanning in patients with recurrent PCa after radical treatment through a systematic literature review. The primary objective after BCR is to locate the site of the recurrent lesion. This study found that 68Ga-PSMA PET/CT also appears to be effective for targeting relapse in patients with very low PSA levels (< 0.5 ng/mL), allowing for early selection of the best treatment strategy. Therefore, the study shows that PSMA ligand PET can change the treatment of PCa patients through accurate diagnosis. In addition, Ceci F et al. (48) evaluated the DR of 68Ga-PSMA PET/CT for PCa recurrence sites, and stratified the population according to different clinical stages of BCR. In a patient-based analysis, 24.7% of cases detected had metastases limited to the pelvic cavity. 28.9% of the patients had at least one distant lesion. The DR of subgroup 1, 2 and 3 were 64.5%, 45.6% and 58.7%, respectively. It was found that 68Ga-PSMA-11 PET/CT has good diagnostic value for PCa patients who failed radical treatment. The impact of 68Ga-PSMA PET diagnosis on the clinical management of PCa has also been studied. Fendler WP et al. (50) described the impact of 68Ga-PSMA PET on the management of recurrent PCa. This study reports the therapeutic changes of 68Ga-PSMA PET in patients with biochemical recurrent PCa after diagnosis. The results showed that 68Ga-PSMA PET could identify the recurrence site of more than half of the patients with BCR of PCa, and translate into changes in treatment plan.

Studies have shown that 68Ga-PSMA PET/CT is also of great diagnostic value in LN staging of PCa patients. Peng L et al. (70) evaluated the diagnostic value of 68Ga-PSMA PET/CT for LN staging in PCa patients through a meta-analysis of diagnostic tests. The results showed a sensitivity of 0.84 and a specificity of 0.95. The results indicate that 68Ga-PSMA PET/CT has a high overall diagnostic value for LN staging in patients with moderate and high risk PCa. Similarly, Petersen LJ et al. (71) compared the primary LN staging of preoperative PSMA PET with histopathology through An expedited systematic review. Eighteen eligible clinical trials involving 969 patients were included in the study. The sensitivity and specificity of PSMA PET was found to be superior to anatomic imaging (CT or MRI). In addition, Tu X et al. (63) evaluated the accuracy of 68Ga-PSMA PET/CT in preoperative LN staging of patients with moderate and high-risk PCa by taking the pathological results of pelvic LN dissection as the reference standard. The results showed that 68Ga-PSMA PET/CT could be used for preoperative LN staging, and patients without LN metastasis had a low misdiagnosis rate. Current guidelines still do not recommend salvage LN dissection (sLND) for PCa patients with BCR due to the low accuracy of existing conventional imaging diagnosis. Kimura S et al. (61) discussed the diagnostic value of 68Ga-PSMA PET for LN metastasis of PCa confirmed by sLND pathology. The results showed that in patients with BCR after radical treatment of PCa, PSMA-PET before sLND was highly accurate and had high positive and negative predictive values. PSMA-PET identifies patients who benefit from sLND and has the potential to perform direct lesion or area dissection. These studies confirm that 68Ga-PSMA PET has higher diagnostic efficacy for LN metastasis of PCa, which may further change the treatment of metastatic PCa.

#### 3.2.2 18F-DCFPyL PET/CT

Currently, conventional imaging examinations such as MRI and CT cannot accurately diagnose LN metastases in initial PCa (72). PSMA PET/CT has been successfully used to stage biochemical recurrent PCa. In addition to the commonly used 68GA-labeled PSMA tracers, there are also 18F-labeled PSMA tracers (18F-DCFPyL, 18F-PSMA-1007). In recent years, low molecular weight radioactive fluorinated radioactive tracers have been gradually applied to PET targeting PSMA. Labeling PSMA tracers with 18F has many advantages, including improved image resolution, extended half-life and improved yield (43, 45). Jansen BHE et al. (52) discussed the diagnostic value of 18F-DCFPyL-PSMA PET/CT in LN staging of primary PCa. The results showed that the sensitivity, specificity, positive predictive value and negative predictive value of 18F-DCFPyL-PSMA PET/CT in detecting pelvic LN metastasis were 41.2%, 94.0%, 53.8% and 90.4%, respectively. In order to more accurately evaluate the detection performance of 18F-PSMA-1007 PET/CT in patients with primary PCa, Huang YT et al. (64) conducted a systematic review and meta-analysis. A total of 12 studies (540 patients) were included. The pooled DR of 18F-PSMA-1007 was 94%, the positive predictive value of 18F-PSMA-1007 was 0.90, the positive predictive value of 18F-PSMA-1007 was 0.94, and the positive predictive value of detection of localized prostate tumors was 0.84. This meta-analysis revealed the superior performance of 18F-PSMA-1007 in detecting localized prostate tumor lesions and regional LN metastases.

Rowe SP et al. (43) evaluated the utility of 18F-DCFPyL PET/CT in patients with metastatic PCa. In this study, 18F-DCFPYL PET/CT imaging was performed in 9 suspected patients with recurrent PCa, 8 patients with metastatic PCa, and 1 patient with BCR. The study compares the detection of suspected metastatic PCa foci between PET and conventional imaging modalities (CIM). A total of 139 metastatic sites were detected by 18 F-DCFPYL PET/CT in 8 patients, while only 45 lesions were detected by CIM. This study demonstrated that 18F-DCFPyL-PSMA PET/CT can detect a large number of suspected PCa sites, many of which are hidden or uncertain in CIM. Similarly, Malaspina S et al. (54) evaluated the tracer uptake and focal detection of the novel radiopharmaceuticals 18F-rhPSMA-7.3 in patients with PCa. It was found that 18F-rhPSMA-7.3 PET/CT was good for the detection of PCa primary and metastatic lesions. These studies provide strong preliminary evidence for the use of second-generation PET imaging agents targeting PSMA in the detection of metastatic PCa, and further support the important value of PET imaging targeting PSMA in PCa.

PSMA PET/CT diagnosis is increasingly used in the treatment of biochemical recurrent PCa worldwide. Dietlein F et al. (44) evaluated the sensitivity of 18F-DCFPyL-PSMA and 68Ga-PSMA to PSA stratification. A total of 191 patients with BCR were scanned with 18F-DCFPyL (62 cases) or 68Ga-PSMA (129 cases). The results of this study confirm that 18F-Dcfpyl-PSMA is not inferior to 68Ga-PSMA, and has the advantage of 18F labeling. In patients with moderately elevated PSA levels after prostatectomy, 18F-DCFPyL-PSMA imaging was also found to improve sensitivity to locate recurrent tumors. In addition, Treglia G et al. (73) conducted a systematic review and meta-analysis of the DR of 18F-PSMA PET/CT in biochemical recurrent PCa. The results showed that 18F labeled PSMA PET/CT had good DR for Biochemical recurrent PCa. Similarly, Song H et al. (51) evaluated the positive rate of 18F-DCFPyL-PSMA PET/CT in diagnosing patients with Biochemical recurrent PCa using a prospective study. The study found that the overall positive rate of 18F-DCFPyL PET/CT was 85%, which increased with the increase of PSA level, and 18F-DCFPyL PET detected more lesions than conventional imaging. These studies indicate that 18F-DCFPyL-PSMA PET/CT is a promising diagnostic tool, with a higher positive rate than currently available imaging methods approved by the US Food and Drug Administration, and has an impact on clinical treatment in 60% of patients.

In addition, another investigator evaluated 18F-PSMA-1007 PET/CT in PCa patients with different serum PSA levels (74). It was found that 18F-PSMA-1007 PET/CT produced 90% to 100% DR in patients with newly diagnosed PCa, with a combined estimate of 94%. The DR of 18F-PSMA-1007 PET/CT for PCa in BCR patients was 47% to 100%, with a combined estimate of 86%. The DR of 18F-PSMA-1007 PET/CT imaging for prostate primary tumor was slightly higher than that for biochemical recurrent tumor. Similarly, Liu X et al. (75) also discussed the application value of 18F-PSMA-1007 PET/CT in PCa patients with different serum PSA levels through systematic evaluation. The results showed that the sensitivity and specificity of 18F-PSMA-1007 PET/CT were 0.934 and 0.453, respectively, and the sensitivity and specificity of single lesion were 0.816 and 0.979, respectively. The diagnostic accuracy of 18F-PSMA-1007 PET/CT was also analyzed as serum PSA levels increased. These studies indicate that 18F-PSMA-1007 PET/CT has high application value in PCa, including primary and BCR tumor.

#### 3.2.3 68Ga-PSMA-11 PET/CT-guided prostate biopsy technique

PSMA PET has been widely accepted as a staging tool for PCa. Recent studies have shown that PSMA PET-guided biopsy (PSMA-PET-TB) has clinical significance in the detection of PCa. Kawada T et al. (76) evaluated the value of PSMA-PET-TB in the diagnosis of csPCa. The results showed that the combined sensitivity and specificity of PSMA-PET-TB for csPCa detection were 0.89 and 0.56 respectively, indicating that PSMA-PET-TB had good diagnostic accuracy for csPCa. Similarly, Zhang LL et al. (59)evaluated the efficacy of 68Ga-PSMA-11 PET/CT-guided biopsy and compared it with transrectal ultrasound-guided puncture biopsy (TRUS-GB) in the diagnosis of csPCa. The results showed that the DR of PCa and csPCa were 43.3% and 40.0% in PSMA PET group and 31.6% and 25.0% in TRUS group, respectively. The DR of PSMA-PET for csPCa (27.02%) was significantly higher than that of TRUS (8.82%), and the difference was statistically significant (*p*<0.05). The results suggest that 68Ga-PSMA-11 PET/CT is a viable imaging technique that can be used as a guide tool for prostate biopsy and may improve the DR of csPCa compared with TRUS-GB.

#### 3.2.4 Comparison of the diagnostic efficacy of different tracer in PCa

The 68Ga-PSMA PET shows good promise in the diagnosis of PCa. However, 68Ga has some disadvantages as a tracer, including a short half-life and non-ideal energy, which has prompted consideration of 18F labeled analogues. 18F-PSMA exhibited a high labeling rate, prominent tumor uptake and rapid, non-urinary excretion. Some scholars have proved that 18F-DCFPyL-PSMA is not inferior to 68Ga-PSMA, and has the advantage of 18F labeling (44). Giesel FL et al. (45) compared the diagnostic efficacy of 18F-PSMA and 68Ga-PSMA as tracers. Three healthy volunteers and 10 patients with high-risk PCa were included in the study. The results showed that 18F-PSMA performed at least as well as 68Ga-PSMA, but its long half-life, superior energy properties and non-urinary excretion overcame some practical limitations of 68Ga-labeled PSMA-targeting tracers.

Choline labeled PET/CT imaging agent is also widely used in the diagnosis of PCa. In order to explore the role of choline as imaging agent in the diagnosis of PCa, Lin CY et al. (77) compared the staging performance of 68Ga-PSMA and F-choline PET/CT imaging for PCa. There was no significant difference between 68Ga-PSMA PET/CT and F-choline PET/CT in staging performance of PCa patients. This study demonstrated that 68Ga-PSMA PET/CT and F-choline PET/CT have high diagnostic efficacy for the accurate staging of PCa patients, and both can be used for the staging of PCa. Subsequently, Moghul M et al. (78) evaluated the application of 68Ga-PSMA PET/CT and F-choline PET/CT in detecting recurrent PCa. The results showed that compared with choline PET/CT, PSMA PET/CT had better performance in detecting recurrence per patient and per lesion, and should be used as the imaging method of first choice in salvage treatment strategy. Similarly, Witkowska-Patena E et al. (49) confirmed that the DR of 18F-PSMA-1007 was higher than that of F-choline PET/CT in patients with early biochemical recurrent PCa. In the former, the total number of lesions was more, the height suggested more lesions, and the unclear lesions were less. In addition, Zhou J et al. (79) systematically evaluated the diagnostic efficacy of SMA-PET/CT, choline -PET/CT, sodium fluoride (NaF) PET/CT, MRI and bone imaging (BS) for bone metastasis of PCa. The results of the study found that PSMA-PET/CT had the highest sensitivity and specificity per patient in detecting bone metastases of PCa. These studies suggest that PET/CT with PSMA has superior diagnostic efficacy than PET/CT with choline.

In addition, National Comprehensive Cancer Network guidelines consider 18F-fluiclovine PET-CT for the location of BCR of PCa after radical prostatectomy, European Association of Urology guidelines recommend the use of PSMA PET-CT. Calais J et al. (47) compared the diagnostic efficacy of 18F-fluiclovine PET/CT and PSMA PET/CT in BCR through prospective clinical studies. The DR of 18F-fluiclovine PET/CT was significantly lower than that of PSMA PET/CT. At the same time, the study also confirmed that for PCa patients with low PSA concentration (≤2.0 ng/mL) and BCR after radical prostatectomy, PSMA marker tracer can be used as the preferred PET tracer in subsequent treatment decision-making, with a high DR. The above studies indicated that 68Ga and 18F are still the main tracers used to mark PSMA because of their superior detection performance.

### 3.3 Comparison of PET imaging strategies targeting PSMA with traditional diagnostic strategies

**Figure 3:**
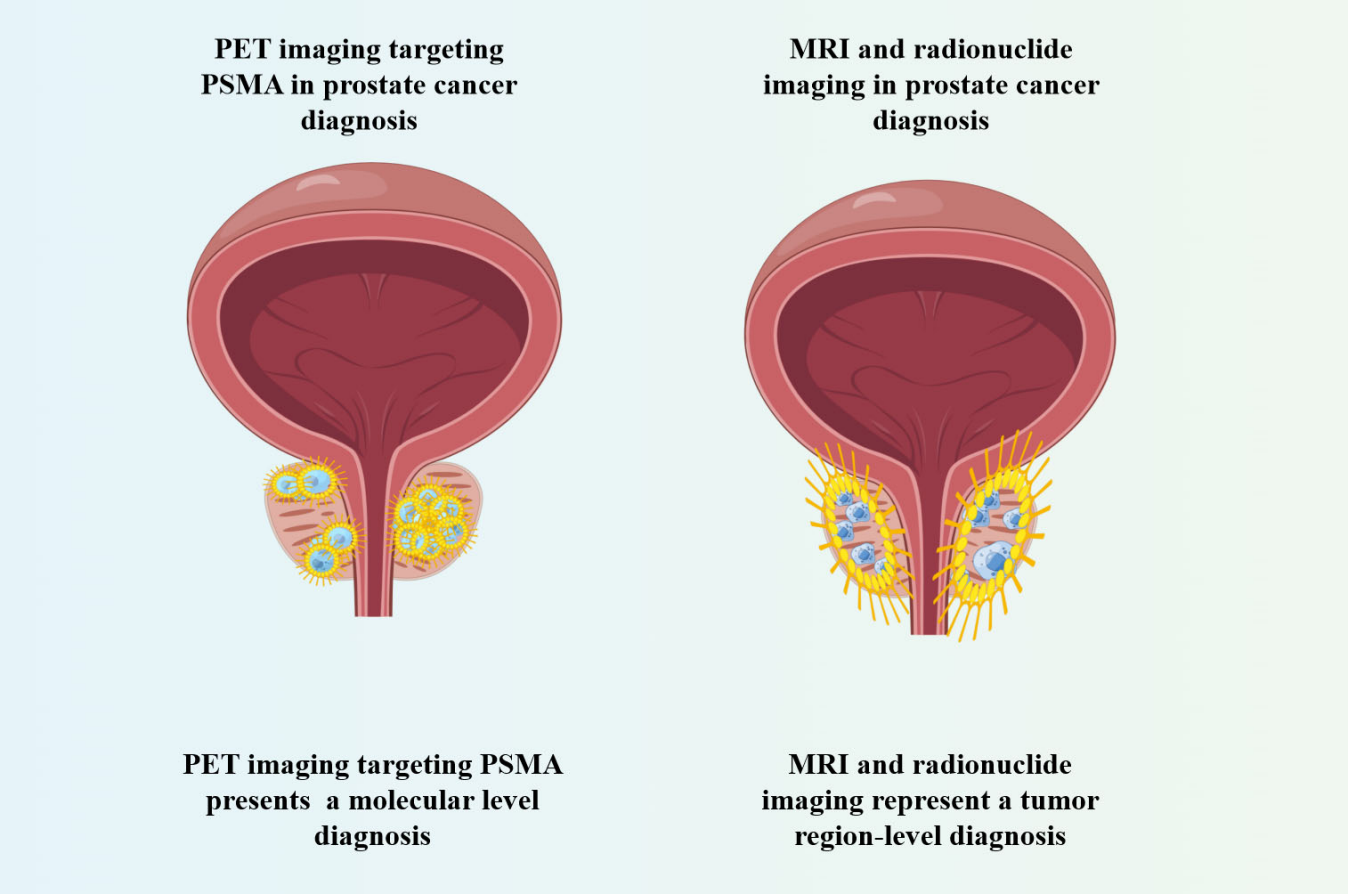
PSMA PET imaging compared with MRI and nuclide imaging in PCa diagnosis.

PET targeting PSMA diagnoses PCa from the molecular level, whereas MRI and nuclide imaging is a diagnosis of tumor areas (**Figure 3**). Due to the excellent diagnostic performance of PET imaging targeting PSMA in PCa, researchers also compared it with traditional diagnostic strategies through a large number of studies (**Table 3**). The results showed that PSMA PET not only had higher sensitivity to detect metastases than mpMRI, but also had higher diagnostic efficacy in N and M stages in moderate and high-risk PCa before treatment. At the same time, it is also found that PSMA PET combined with CT or mpMRI can improve the diagnostic efficiency of PCa, especially PSMA PET/CT is widely used in the clinical diagnosis of PCa.

**Table 3:** Comparative studies on the efficacy of targeted PSMA diagnostic strategies and traditional diagnostic strategies in PCa diagnosis.

Interventions	Type of Study	Objectives	Number of Studies and/or Patients	Conclusion	References
BS and 68Ga-PSMA PET	Retrospective cohort study	This study compared the diagnostic efficacy of 68Ga-PSMA PET and (99m) Tc bone imaging for bone metastases in PCa.	126 patients	68Ga-PSMA PET is superior to BS in the determination of global bone involvement in PCa patients.	Pyka T et al.2016 (55)
mpMRI and 68Ga-PSMA PET	Prospective non-randomized controlled study	The true sensitivity and specificity of prostate mpMRI and 68Ga-PSMA PET for detecting and locating tumor lesions in the prostate were compared.	20 patients	A significant proportion of cancers may be missed and underestimated by both imaging methods.	Rhee H et al.2016 (56)
68Ga-PSMA PET/CT and mpMRI	Prospective non-randomized controlled study	The value of 68Ga-PSMA PET/CT and mpMRI for staging of high-risk PCa was compared.	36 patients	68Ga-PSMA PET/CT can be used for LN and metastatic staging of high-risk PCa.	Tulsyan S, et al.2017 (57)
PSMA PET/CT	Retrospective study	This study investigated the value of preoperative PET/CT staging of PSMA in prostate treatment.	142 patients	Extrprostatic lesions detected by preoperative PET staging of PSMA were an independent risk factor for poor outcome of PCa surgery.	Nandurkar R et al.2019 (58)
68Ga-PSMA-11 PET/CT	Prospective controlled study	This study evaluated the efficacy of 68Ga-PSMA-11 PET/CT-guided prostate biopsy.	120 patients	68Ga-PSMA-11 PET/CT can be used as a guide tool for prostate biopsy.	Zhang LL et al.2021 (59)
68Ga-PSMA-11 PET/CT	Prospective non-randomized controlled study	This study investigated the application value of 68Ga-PSMA-11 PET/CT in the N and M stages of patients with moderate and high risk PCa.	81 patients	68Ga-PSMA-11 PET/CT has high diagnostic efficacy in N and M stages of middle and high risk PCa patients, and is superior to pelvic mpMRI in detecting local regional LN metastasis.	Szigeti F et al.2022 (60)

#### 3.3.1 mpMRI and PSMA-PET

In recent years, mpMRI has become an important means of PCa diagnosis due to its value in diagnosis and staging of PCa. Rhee H et al. (56) compared the true sensitivity and specificity of mpMRI and PSMA PET for detecting and locating tumor lesions in the PCa through a prospective clinical trial. All patients underwent mpMRI and PSMA-PET scanning before surgery, and were directly compared with pathological sections. The results found that a significant proportion of cancers with both imaging methods were likely to be missed or underestimated. PSMA-PET combined with mpMRI can improve the local staging of patients undergoing radical retropubic prostatic cancer.

In a systematic review and meta-analysis, 68Ga-PSMA PET compared with mpMRI in the diagnosis of LN metastasis stage in PCa (80). This study summarized the diagnostic efficacy of 68Ga-PSMA PET and mpMRI for LN metastasis of middle and high risk PCa. The results showed that the sensitivity of 68Ga-PSMA PET to detect metastases was higher than that of mpMRI, and the specificity was slightly different in moderate and high-risk PCa before treatment. The area under the SROC curve suggested that 68Ga-PSMA PET was a more effective predictor of LN metastasis before radical surgery. Secondly, Tulsyan S et al. (57) compared the value of 68Ga-PSMA-11 PET and mpMRI in the staging of high-risk PCa. This prospective non-randomized controlled study included 36 patients at high risk for PCa. The results show that 68Ga-PSMA PET/CT can be used for LN and metastasis staging of high-risk PCa, but its value for staging prostate disease is limited. In addition, a meta-analysis compared the diagnostic efficacy of 68Ga-PSMA-11 PET/CT versus mpMRI in preoperative pelvic LN staging in patients with PCa (81). Nine studies (640 patients) were included in the study. The results showed that 68Ga-PSMA-11 PET/CT showed higher sensitivity and diagnostic accuracy in detecting pelvic LN staging in PCa patients compared with mpMRI. Szigeti F et al. (60) compared the performance of 68Ga-PSMA-11 PET/CT and mpMRI in detecting local LN metastases and intraprostate tumor lesions. The results of this study confirmed that 68Ga-PSMA-11 PET/CT has high diagnostic efficacy in N and M stages of medium-high risk PCa patients, and is superior to pelvic mpMRI in detecting local regional LN metastasis. These studies indicate that 68Ga-PSMA-11 PET/CT is superior to mpMRI in the diagnosis of LN metastasis stage of PCa, and has important diagnostic value for the clinical management and detection of metastatic PCa.

#### 3.3.2 68Ga-PSMA PET and BS

BS is the most widely used method for early assessment of bone metastases in PCa (21). BS is a highly sensitive imaging method with low specificity. Numerous studies have been conducted on the ability of 68Ga-PSMA PET/CT to detect malignant bone lesions and whether this method is superior to existing BS methods. BS using (99m) TC-labeled methylene bisphosphate [(99m) Tc-MDP] remains the recommended imaging mode for detecting bone metastases in patients with PCa. However, PET/CT using PSMA ligands is increasingly being considered as a means of assessing the extent of disease in patients with PCa, including as a possible independent test in high-risk patients.

It was found that the diagnostic efficacy of PSMA-PET/CT was significantly better than BS in the diagnosis of bone metastasis of PCa (79). Pyka T et al. (55) compared the diagnostic efficacy of 68Ga-PSMA PET and BS in bone metastasis of PCa. This study retrospectively analyzed the diagnostic value of BS and 68Ga-PSMA PET in 126 patients with PCa. BS, PET, other imaging and follow-up data were used to determine the best value for comparison. The results of this study showed that 68Ga-PSMA PET was superior to plane BS in detecting the affected bone area and determining the overall bone involvement in PCa patients. Similarly, Zacho HD et al. (82) explored the diagnostic value of 68Ga-PSMA PET/CT for bone metastases in PCa. A total of 37 studies were analyzed in this systematic review, which found that 68Ga-PSMA PET/CT showed more lesions than bone imaging, and in particular improved the diagnostic efficacy of mCRPC compared to bone imaging. Gege Zhao et al. (83) compared the diagnostic efficacy of 68Ga-PSMA-11 PET/CT with (99m)Tc-MDP BS in bone metastases of PCa through a meta-analysis. The combined sensitivity and specificity of 68Ga-PSMA-11 PET/CT were 98% and 97%, and that of (99m) Tc-MDP BS were 83% and 68%, respectively. The results showed that 68Ga-PSMA-11 PET/CT was superior to (99m) Tc-MDP BS in the diagnosis of PCa bone metastases. In addition, Zhao R et al. (84) also evaluated the value of 68Ga-PSMA-PET/CT and BS in the clinical diagnosis of PCa from the perspective of evidence-based medicine. By comparing the diagnostic results of 68Ga-PSMA-PET/CT and BS, the study found that 68Ga-PSMA-PET/CT has higher sensitivity and specificity than BS, and has higher diagnostic efficacy for bone metastasis of PCa, which is worthy of clinical application. These studies indicate that 68Ga-PSMA PET/CT has a broad application prospect in the diagnosis of PCa.

## 4 Discussion

In recent years, mpMRI has become known for its high spatial resolution and DR in the diagnosis of PCa (19). However, mpMRI cannot accurately diagnose the pathological staging of advanced PCa, especially metastatic LN staging. PSMA is of great value in the diagnosis of PCa because of its specific expression in PCa. Currently, researchers have developed PET targeting PSMA, and numerous studies have demonstrated that compared with traditional imaging methods, it not only has a higher tumor DR in recurrent PCa, but also has a higher diagnostic efficacy in LN staging of advanced PCa. Secondly, studies have shown that hybrid PET/CT and PET/MRI targeting PSMA are more accurate in the diagnosis of metastatic and recurrent PCa (85–87). In addition, invasive biopsy of PCa has a significant false negative rate (88), and studies have shown that guided biopsy of PCa by PSMA PET/CT can significantly improve the DR (59, 76). Therefore, PSMA PET/CT is of great value in the diagnosis of PCa.

PSMA PET is now part of international guidelines for PCa diagnosis and has received its first regulatory approval (89). Several radioactive tracers of PSMA, including 18F and 68Ga, have been used for PET imaging (90–92). A large number of studies have shown that PSMA PET imaging has higher accuracy, specificity and sensitivity than traditional imaging (55–57, 80, 82). At present, 68Ga tracer is the most widely used PSMA targeting radiopharmaceutical. According to FDA approval, 68Ga-PSMA-11 is intended for patients with suspected PCa metastases that are likely to be cured with surgery or radiation, and for patients with suspected recurrence of PCa. Furthermore, 68ga-labeled tracers could detect more lesions. However, 18F radiolabeled PSMA targeting agents have several advantages over 68Ga radiolabeled agents in PET imaging, including a longer radiotracer half-life and better PET image resolution (92, 93). On the other hand, 18F-labeled PSMA PET imaging has shown very promising results in primary tumor detection and T staging. Since there is no excretion of urine, it seems to be advantageous in the pelvic area. The above results indicate that the diagnostic efficacy of PET imaging techniques with different PSMAs targeting may vary, and appropriate tracers should be selected according to the tumor characteristics of patients in clinical application.

The ultimate goal of PSMA PET/CT in PCa imaging is to optimize clinical management and thereby improve patient outcomes. Notably, studies have shown that targeted imaging of PSMA can alter the management of 12.6-30% of patients (94, 95). In recent years, radioactive tracers based on PSMA have been studied most extensively. Studies have shown that PSMA based imaging strategies affect treatment decisions for biochemical recurrent PCa. Ongoing prospective studies will begin to elucidate the impact of PSMA imaging on patient outcomes. In addition, we found that PSMA PET/CT also has a higher lesion DR in patients with low PSA level, indicating that this technique as a molecule-targeted diagnostic strategy is applicable to a wide population of PCa patients. As is known to all, PSMA PET/CT is currently mainly used in the detection of recurrent diseases, and rarely used in the staging of primary tumors (96, 97). Therefore, it is not yet a standard procedure in the diagnosis of the primary stage of PCa. The FDA recently approved the use of PSMA PET/CT in the primary stage diagnosis of PCa, but it is still not explicitly mentioned in the guidelines. Further studies are necessary to evaluate the long-term effects of PSMA-targeted PET imaging to determine its role in the primary stages of PCa. PSMA PET/CT is expected to change the clinical management strategy of patients by detecting the progress of PCa in the whole stage, so as to achieve a better clinical outcome for patients.

At present, PCa targeting PSMA has achieved certain results in the diagnosis of PCa. However, due to the influence of current device performance and tumor heterogeneity, it has not been widely promoted in clinical practice. First, due to the limited spatial resolution of the imaging equipment and high background activity in the pelvic region, tumor metastases and small LN metastases may present suspicious results. Secondly, due to the heterogeneity of prostate tumors, some patients showed low expression of PSMA on the tumor cell surface (98, 99). The diagnostic efficacy of PSMA PET/CT for these patients will be reduced. This is also a common problem faced by all molecular targeted diagnostics and therapies. It is believed that with the rapid development of PET imaging technology and molecular targeting technology, the above influencing factors will be overcome.

## 5 Conclusions

To sum up, the progression of PCa varies greatly among different individuals. Even after standardized treatment, some patients will still progress to mCRPC, which makes it particularly important to adopt better clinical management of PCa through precise diagnostic strategies. A large number of studies have confirmed that PET targeting PSMA not only has a higher tumor DR in primary PCa, but also has a higher diagnostic efficacy in advanced PCa metastases and LN staging compared with traditional imaging methods. Therefore, PET targeting PSMA is worthy of clinical promotion in primary, metastatic and recurrent PCa.

## Author contributions

YL, ZZ and PS are responsible for the conception,execution and amendment of this article, and all authors are responsible for the production of pictures and language polishing. All authors contributed to the article and approved the submitted version.
